# Enlarged Pericarotid Lymph Nodes Suggest Recent Ischemic Symptoms in Patients with Carotid Atherosclerosis

**DOI:** 10.3389/fimmu.2022.900642

**Published:** 2022-07-12

**Authors:** Tao Sun, Fei Wang, Yiming He, Bo Mao, Mengtao Han, Han Liu, Peng Zhao, Xingang Li, Donghai Wang

**Affiliations:** ^1^ Department of Neurosurgery, Qilu Hospital of Shandong University, Cheeloo College of Medicine, Shandong University, Jinan, China; ^2^ Institute of Brain and Brain-Inspired Science, Cheeloo College of Medicine, Shandong University, Jinan, China; ^3^ Department of Critical Care Medicine, Qilu Hospital of Shandong University, Cheeloo College of Medicine, Shandong University, Jinan, China

**Keywords:** carotid atherosclerosis, pericarotid lymph nodes, ischemic symptoms, adaptive immune response, plaque instability

## Abstract

Atherosclerosis is a chronic inflammatory disease closely associated with immunological activity. Lymph nodes (LNs) are essential secondary lymphoid organs, in which complex immune responses occur. Enlarged LNs are commonly observed around inflamed tissues or tumors; however, their role in atherosclerosis is not well understood. We hypothesized that enlarged pericarotid LNs would be present in symptomatic patients with carotid atherosclerosis. Therefore, we recorded the size of LNs around the carotid artery during surgery in patients undergoing carotid endarterectomy (CEA) for carotid atherosclerotic stenosis. Patients were stratified by enlarged LNs, defined as a diameter ≥ 10mm in the transverse diameters. Demographic and clinical data of participants were measured and analyzed. Hematoxylin and eosin (H&E), Sirius red, DAB-enhanced Perls’ Prussian blue, alizarin red, and immunohistochemistry (IHC) staining were performed for composition identification of plaques or LNs. Symptomatic patients were defined as those presenting with an ipsilateral cerebral ischemic event. Compared with patients with non-enlarged LNs, patients with enlarged LNs were more likely to be symptomatic (22/32, 68.8% versus 9/40, 22.5%, *P* < 0.001) and use calcium channel blocker drugs (17/32, 53.1% versus 10/40, 25%, *P*=0.014). In addition, they showed lower body mass index (mean ± SD: 24.00 ± 2.66 versus 25.34 ± 2.56 kg/m^2^, *P*=0.034), lower weight (median [interquartile range]: 64 [60.00-76.00] versus 72.5 [65.00-77.50] Kg, *P* = 0.046) and higher diastolic blood pressure (mean ± SD: 78.94 ± 9.30 versus 73.93 ± 8.84 mmHg, *P* = 0.022). The plague from patients with enlarged LNs exhibited a lower relative percentage of fibrous tissue (29.49 ± 10.73% versus 34.62 ± 10.33%, *P* = 0.041). The enlarged LNs remained oval-shaped by visual inspection. Compared to non-enlarged LNs, the predominant changes in enlarged LNs were atrophic lymphatic sinuses and dilated LNs parenchyma. Enlarged LNs contained more germinal centers and lymphocytes. In conclusion, symptomatic patients with carotid atherosclerosis have enlarged pericarotid LNs. The current study supports the conclusion that enlarged LNs with an activated and enhanced adaptive immune response may indicate plaque instability. Pericarotid LNs will be a promising marker of plaque stability and may be a potential therapeutic target in patients with carotid atherosclerosis.

## Introduction

Atherosclerosis is a chronic inflammatory disease closely related to intense immunological activity (1, 2). Accumulation of lipids, vascular inflammation, immune cell activation, foam cell formation, cell apoptosis, and necrosis play important roles in both formation and development of atherosclerotic plaques (3). As the lesion progresses, unstable atherosclerotic plaques tend to rupture and cause ischemic stroke.

As early as 1980, Lemole et al. identified lymphstasis as a critical factor in the genesis of arteriosclerosis (4), but the role of the lymphatic network system, which carries a wide variety of immune cells, in the development of atherosclerosis has only recently been explored. Martel et al. introduced the concept of macrophage reverse cholesterol transport (mRCT) and identified the role of lymphatic vessels in reverse cholesterol transport (5). Subsequent studies have also shown that the lymphatic network is an important way of mobilizing cholesterol from the artery walls (6–8).

Lymph nodes (LNs) are essential secondary lymphoid organs that play an important role in immune responses (9). Enlarged LNs, which are commonly observed around inflamed tissues or tumors, usually indicate a strong inflammatory and immune responses (10, 11). Particular antigens, bacteria and viruses flowing through draining LNs can be effectively cleared, indicating that LNs are an effective filter (9, 12). LNs draining lymph from the arteries may play a more complex role in the course of atherosclerosis. Ox-low-density lipoproteins (LDLs) deposited in plaques can be taken up by DCs and then presented to T cells in draining LNs, thus initiating adaptive immune (2). However, in a healthy artery wall, resident DCs usually exert immune tolerance by silencing T cells (13).

**Figure 1:**
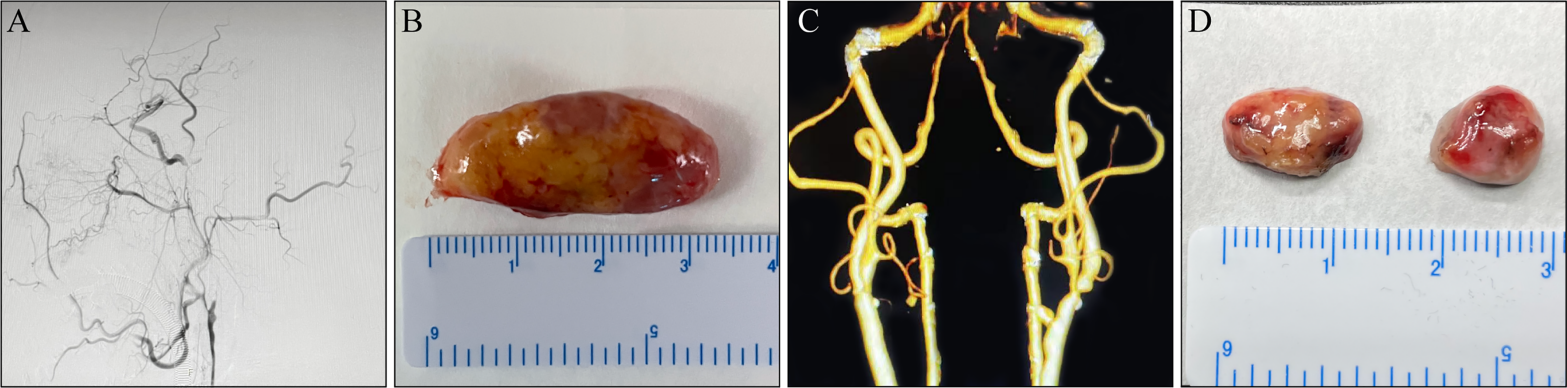
Macroscopic lymph nodes (LNs) morphology. **(A, B)** A 65-year-old man complaining of alalia for 1 month was hospitalized. **(A)** Digital subtraction angiogram (DSA) before carotid endarterectomy (CEA) revealed a left internal carotid artery (ICA) with complete occlusion. **(B)** An enlarged LN around the carotid artery. **(C, D)** A 74-year-old man was admitted to hospital owing to “carotid artery stenosis found on physical examination for 3 months”. **(C)** DSA before CEA revealed a left ICA with severe stenosis. **(D)** Non-enlarged LNs around the carotid artery.

Recent single-cell sequencing has uncovered a new function of LN endothelial cells in scavenging LDLs (14); During the carotid endarterectomy (CEA), enlarged pericarotid LNs were observed in some patients (**Figure 1B**). In this study, we aimed to explore the relationship between enlarged LNs and recent ischemic symptoms, which helped us improve our understanding of the predictors of plaque vulnerability and better treat this high-risk group of patients with carotid atherosclerosis.

## Materials and Methods

### Patients Selection

This study was approved by the ethics committee of our hospital. Informed consent was obtained from all patients prior to the procedure. Patients undergoing CEA for carotid atherosclerotic stenosis or occlusion in our department were enrolled consecutively in the study between December 2020 and January 2022. All patients were clearly diagnosed and other etiologies were excluded by Doppler ultrasound, computed tomography angiography (CTA), magnetic resonance imaging or angiography, and digital subtraction angiography (DSA). The minimum age limit was 18. Patients with neck tumors, a history of neck radiation, lymphoma, and lymph node tuberculosis were excluded. LN size around the carotid artery, including longitudinal and transversal diameters, was recorded during the operation. If there is more than one lymph node around the carotid artery, the largest one is recorded. Enlarged LNs were defined as those with transverse diameters ≥ 10 mm (15, 16). Symptomatic patients had a history of amaurosis fugax, transient ischemic attack, or ischemic stroke ipsilateral to the extracted plaques within six months before the endarterectomy procedure. Conversely, asymptomatic patients did not have a history of cerebrovascular events (17). Data on venous blood samples, and the clinical and demographic characteristics of the participants were gathered from patients before CEA.

### Plaque Specimens

For this study, 72 formalin-fixed paraffin-embedded blocks of carotid artery specimens met the inclusion criteria. All samples exhibited morphological characteristics of atherosclerotic plaques such as a necrotic core, connective tissue, and the presence of at least a portion of the fibrous cap in sectioned slides. Hematoxylin and eosin (H&E) staining was performed according to standard procedures (18). Macrophages were identified in consecutive sections by immunohistochemistry (IHC) using primary antibodies against CD68 (Cell Signaling Technology, 76437T) (19). Picrosirius red staining was used to detect collagen fibers (19). DAB-enhanced Perls’ Prussian blue staining was used to evaluate the presence of intraplaque hemorrhage (19). Alizarin red staining was used to evaluate calcium deposition (19). Quantification of staining was documented as the threshold area divided by the lesion area using ImageJ software.

### LN Specimens

H&E staining and IHC were performed on formalin-fixed and paraffin-embedded LNs, which included nine enlarged and five non-enlarged LNs. All LNs were dissected transversely in the middle of the longitudinal axis and serially sectioned. The primary antibodies used were anti-CD20 (Cell Signaling Technology, 48750T, 1: 100) for identifying B cells, anti-CD68 (Cell Signaling Technology, 76437T) for identifying macrophages, anti-CD3 (Servicebio, GB11014) for identifying T cells, and anti-Ki67(Cell Signaling Technology, 9449T) for identifying proliferating cells (18). Diluents without primary antibodies were used as negative controls. Staining was visualized using the Dako REAL™ EnVision™ Detection System followed by counterstaining with hematoxylin. Images were captured using a digital camera under a light microscope (VS120; Olympus). The number of germinal centers (GCs) observed was counted in one HE-stained section. The proportions of CD68- and Ki67-positive cells in the LNs were calculated as positive cells versus total cells in at least five randomly selected areas under ×1000 magnification of microscopic fields.

### Data Analysis

In this study, all statistical analyses were performed using the SPSS software version (version 25.0; SPSS, Inc., Chicago, IL, USA). Normally distributed continuous variables were expressed as the mean ± standard deviation, and were analyzed using the Student’s t-test. Abnormally distributed continuous variables were expressed as median (interquartile range [IQR]) and analyzed by the Mann-Whitney U test. Categorical variables were described as percentages, and were analyzed using the chi-square test or Fisher’s exact test. Statistical significance was set at *P* < 0.05.

## Results

### Clinical and Biochemical Characteristics of Patients

Baseline clinical, biochemical, and demographic characteristics of the study participants are shown in **Table 1**. No significant differences in age, sex, systolic blood pressure, pulse pressure, serum low-density lipoprotein cholesterol, cholesterol, or triglyceride levels were found between patients with enlarged and non-enlarged LNs (*P* > 0.05). Compared with patients without enlarged LNs, patients with enlarged LNs were more likely to be symptomatic (68.8% vs. 22.5%, *P* < 0.001) and more likely to use calcium channel blocker drugs (53.1% vs. 25%, *P* = 0.014). In addition, they showed lower BMI values (mean ± SD: 24.00 ± 2.66 versus 25.34 ± 2.56 kg/m^2^, *P* = 0.034), lower weight (median [interquartile range]: 64 [60.00-76.00] versus 72.5 [65.00-77.50] Kg, *P* = 0.046), and higher diastolic blood pressure (mean ± SD: 78.94 ± 9.30 versus 73.93 ± 8.84 mmHg, *P* = 0.022).

**Table 1:** Baseline Characteristics of Study Participants.

Demographic, clinical, and laboratory items	Enlarged LN group	Non- enlarged LN group	*P* value (<0.05)
No. patients, n	32	40	–
Age, y	64.50 ± 7.39	65.70 ± 6.64	0.471
Sex, male	28 (87.5%)	33 (82.5%)	0.798
Symptom, n	22 (68.8%)	9 (22.5%)	< 0.001
BMI, kg/m^2^	24.00 ± 2.66	25.34 ± 2.56	0.034
Weight, kg	64 (60.00-76.00)	72.5 (65.00-77.50)	0.046
Systolic blood pressure, mmHg	140.66 ± 18.10	139.95 ± 16.71	0.864
Diastolic blood pressure, mmHg	78.94 ± 9.30	73.93 ± 8.84	0.022
Pulse pressure, mmHg	61.72 ± 15.60	66.03 ± 18.11	0.290
Total cholesterol, mmol/L	3.58 ± 0.64	3.55 ± 0.86	0.891
HDL-C, mmol/L	1.07 (0.88-1.15)	0.98 (0.82-1.16)	0.264
LDL-C, mmol/L	1.98 ± 0.52	1.92 ± 0.60	0.648
Triglyceride, mmol/L	1.26 (0.96-1.55)	1.26 (0.97-1.60)	0.856
hCY, μmol/L	14.10 (11.00-17.53)	12.15 (9.50-16.10)	0.087
Component C1q, mg/L	157.16 ± 24.93	162.17 ± 26.33	0.414
Serum glucose, mmol/L	5.25 ± 0.74	5.32 ± 0.90	0.717
Serum uric acid, μmol/L	331.28 ± 75.29	344.33 ± 77.28	0.474
Diabetes mellitus, n	3 (10.7%)	7 (17.5%)	0.667
Hypertension, n	26 (81.3%)	25 (62.5%)	0.082
Coronary heart disease, n	11 (34.4%)	11 (27.5%)	0.529
History of smoking, n	19 (59.4%)	19 (47.5%)	0.316
History of drinking, n	16 (50.0%)	15 (37.5%)	0.287
Use of aspirin, n	9 (28.1%)	14 (35.0%)	0.534
Use of clopidogrel, n	5 (15.6%)	3 (7.5%)	0.276
Use of beta-blockers, n	2 (6.3%)	7 (17.5%)	0.282
Use of calcium channel blockers, n	17 (53.1%)	10 (25.0%)	0.014
Use of ACEI or ARB, n	5 (15.6%)	12 (30.0%)	0.154
Use of statins, n	9 (28.1%)	10 (25.0%)	0.765
Stenosis of carotid artery, n (50-69%/70%-99%/100%)	0/28/4	2/34/4	0.725

Data presented as mean ± standard deviation or median (IQR) based on normality of continuous variables. Data presented as n (%) for dichotomous or categorical variables. LN, lymph node; BMI, body mass index; IQR, interquartile range; HDL-C, high-density lipoproteins cholesterol; LDL-C, low density lipoprotein cholesterol; hCY, homocysteine; ACEI, angiotensin converting enzyme inhibitor; ARB, angiotensin receptor blocker.

### Histological Characteristics of Plaque Samples

**Figure 2:**
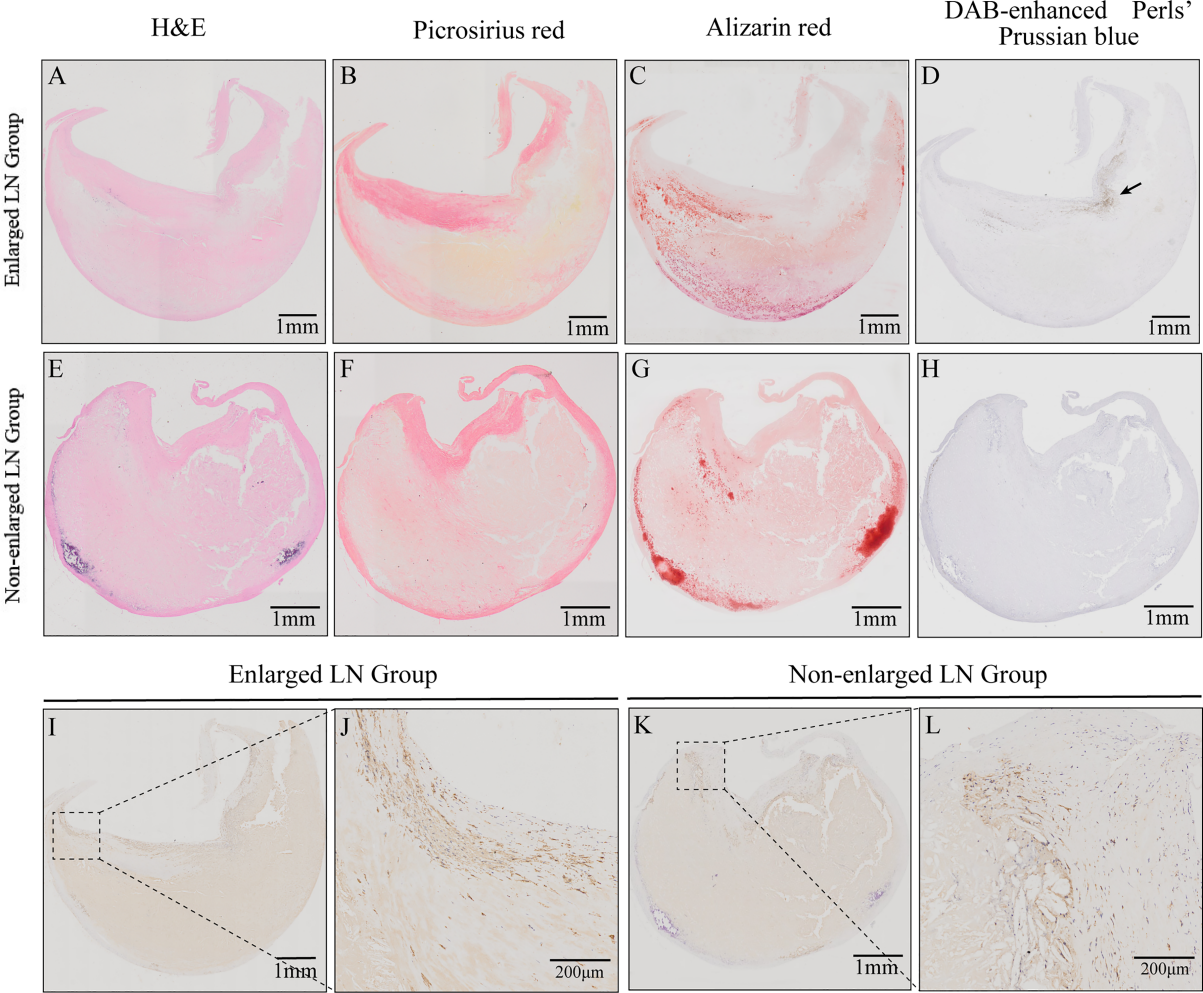
Histological characteristics of carotid atherosclerotic plaques from patients with enlarged or non-enlarged lymph nodes (LNs). **(A, E)** Hematoxylin and eosin (H&E) staining. **(B, F)** Relative content of collagen fibers and thickness of fiber cap. **(C, G)** Relative content of calcification and calcium morphology. **(D, H)** Intraplaque hemorrhage (arrow). **(I–L)** Immunohistochemistry (IHC) staining revealed macrophages and their infiltration in the shoulder regions.

The plagues from patients with enlarged LNs exhibited a lower relative percentage of fibrous tissue (29.49 ± 10.73% vs. 34.62 ± 10.33%, *P* = 0.041, **Table 2**). There were no significant differences in incidences of intraplaque hemorrhage or plaque calcification between patients with enlarged and non-enlarged LNs (*P* > 0.05, **Table 2**). Macrophage infiltration was present in all plaques, mainly in the shoulder region (**Figures 2I–L**). No difference was observed in the relative percentage of the histological components of atherosclerosis including fibrosis, plaque hemorrhage, and calcium between the two groups (*P* > 0.05, **Table 2**).

**Table 2:** Histological Characteristics of Plaques.

Histological characteristics	Enlarged LN group	Non-enlarged LN group	*P* value (<0.05)
Number, n	32	40	–
Fibrous tissue relative percentage, %	29.49 ± 10.73	34.62 ± 10.33	0.041
Intraplaque hemorrhage, n (%)	18 (56.3%)	24 (60.0%)	0.748
Plaque hemorrhage relative percentage, %	0.15 (0.00-0.87)	0.45 (0.00-1.00)	0.467
Calcification, n (%)	27 (84.4%)	36 (90.0%)	0.720
Calcium relative percentage, %	6.65 (1.28-8.35)	7.25 (2.30-11.38)	0.284

Data presented as mean ± standard deviation or median (IQR) based on normality of continuous variables. Data presented as n (%) for dichotomous or categorical variables. LN, lymph node; IQR, interquartile range.

### Morphology and Histological Characteristics of LN Samples

**Figure 3:**
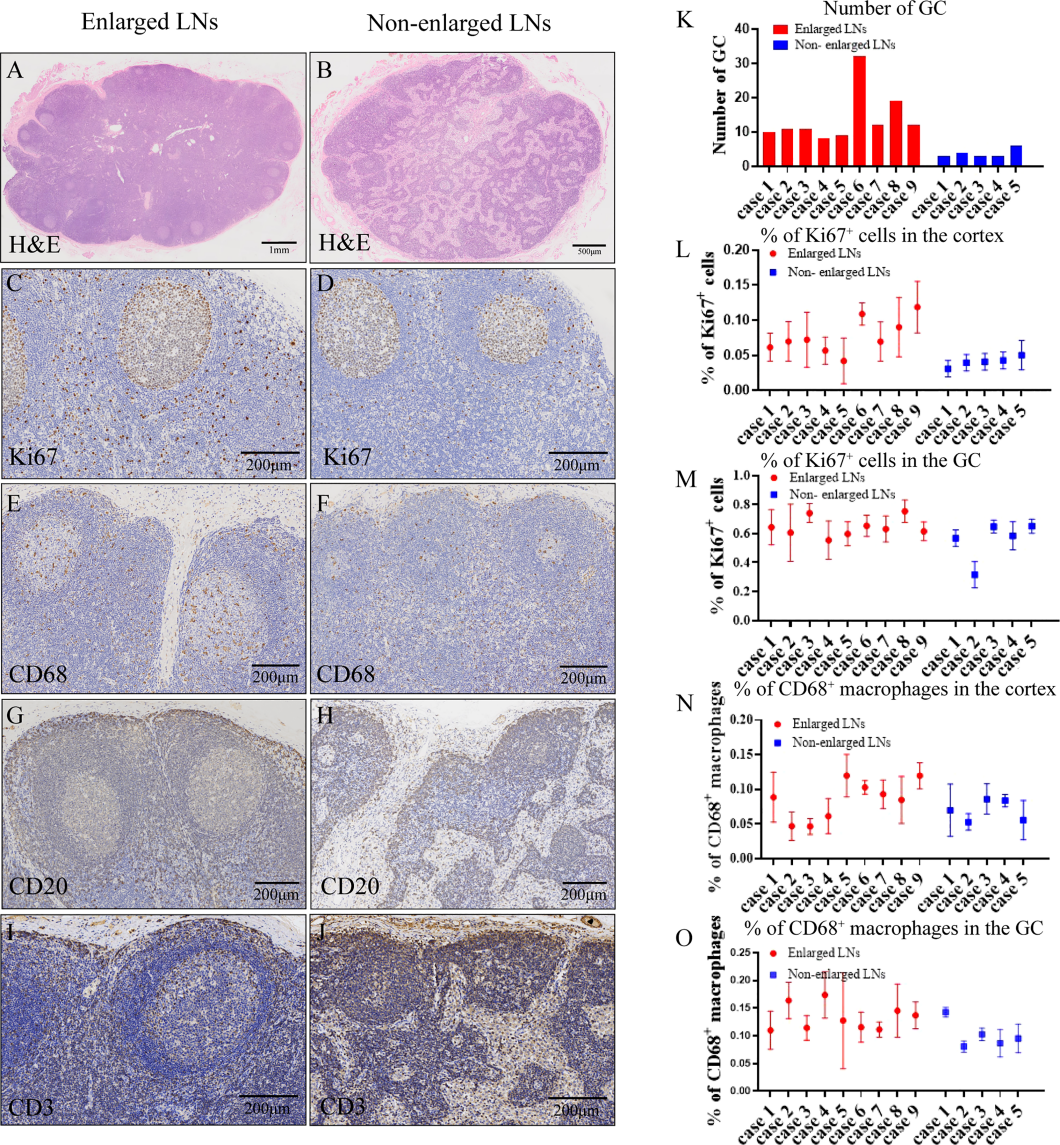
Histological characteristics of lymph nodes. **(A, B, K)** Hematoxylin and eosin (H&E) staining **(A, B)** showing microscopic characteristics of lymph nodes (LNs) and number **(K)** of germinal centers (GCs) in one HE-stained section. **(C, D, L, M)** Immunohistochemistry (IHC) staining of Ki67 **(C, D)** and proportion of Ki67-positive cells **(L, M)** in the cortex or GC of enlarged and non-enlarged LNs. **(E, F, N, O)** IHC staining of CD68 **(E, F)** and proportion of CD68-positive macrophages **(N, O)** in the cortex or GC of enlarged and non-enlarged LNs. **(G, H)** IHC staining showing the increased CD20-positive B cells in the enlarged LNs. **(I, J)** IHC staining showing the increased CD3-positive T cells in the enlarged LNs.

Morphologically, the enlarged LNs were oval (transverse diameter, mean ± SD:13.12 ± 2.23 mm), whereas the non-enlarged LNs (transverse diameter, mean ± SD: 7.28 ± 1.34 mm) were round or oval (**Figure 1**). The predominant changes in enlarged LNs were atrophic lymphatic sinuses and dilated parenchyma (**Figures 3A, B**). Compared with non-enlarged LNs, enlarged LNs contained more and larger GCs (**Figures 3A, B, K**). The proportion of Ki67 positive cells was increased in the cortex of enlarged LNs compared with that of non-enlarged LNs (**Figures 3C, D, L**). The proportion of Ki67-positive cells was similar regarding GCs between the two (**Figures 3C, D, M**). CD68-positive macrophages were mainly infiltrated in the lymphoid sinus (**Figures 3E, F**). The proportion of CD68-positive macrophages showed no apparent changes in the cortex excluding sinus of enlarged LNs, but was increased slightly in the GCs (**Figures 3E, F, N, O**). The number of CD20-positive B cells and CD3-positive T cells increased in enlarged LNs (**Figures 3G–J**).

## Discussion

Ischemic stroke is a common cause of significant morbidity, mortality, and disability-adjusted life-years (DALYs) worldwide (20). The underlying pathological process is atherosclerosis, a chronic disorder of the intimal layer of large-and medium-sized arteries associated with inflammation and immunity (21). Significant stenosis of the carotid artery, rupture of unstable plaques, and subsequent thrombosis result in transient ischemic attacks (TIAs) or ischemic strokes (21, 22). Currently, the percentage of carotid stenosis based on angiographic measurements remains a major criterion for risk stratification in patients with carotid artery stenosis (23). This method has also been validated in randomized clinical trials (RCT) and meta-analyses, which demonstrated that CEAs reduce the risk of future stroke in symptomatic patients with carotid stenosis (24, 25). However, this method does not provide information about plaque composition, plaque stability, inflammation, intraplaque hemorrhage (IPH), ulceration, and calcification. It is widely recognized that plaque vulnerability is more important than degree of stenosis in evaluating the risk of stroke. Many attempts have been made to identify patients with unstable atherosclerotic plaques to prevent stroke. Some researchers are developing advanced imaging systems that can identify plaque components, such as high-resolution magnetic resonance imaging, ultrasonography, and CT angiography (22, 23). Others search for plasma biomarkers such as C-reactive protein (CRP), interleukin-6 and P-selectin (26). Clinical studies and meta-analyses have confirmed the feasibility of these markers and their effectiveness in risk assessment. Nevertheless, peripheral carotid LNs, which are closely associated with plaque inflammation and immune responses, have received little attention. Our study focused on lymph nodes around the atherosclerotic carotid artery. We found that enlarged LNs around the carotid artery in patients with carotid atherosclerosis suggest recent ischemic symptoms.

LNs are mainly composed of the cortex, paracortical cortex and medulla. These are indispensable secondary lymphoid organs. They also generate a highly specialized microenvironment in response to effective immune responses and play an important role in immune initiation and efficacy (14, 27–29). In the body’s response to diseases, such as infections and tumors, enlarged LNs can be seen as a sign that the body’s immune function is activated or expanded. Pericarotid LNs, which drain lymph fluid from the plaque, are likely to indicate plaque inflammation and a strong immune response, thereby reflecting plaque instability. The results of our study support this hypothesis. Among patients with carotid atherosclerosis, we found that a majority of patients with recent ischemic symptoms, which suggest plaque vulnerability, had enlarged pericarotid LNs, and there was a strong statistical significance between the two (*P* < 0.001). Our analysis of plaque composition also supported this view. We found that plagues from patients with non-enlarged LNs were more likely to be fibrous (**Table 2**), suggesting that these plaques were more stable (30). Previous animal studies have reported the expansion of LNs draining the atherosclerotic aorta in aged atherosclerotic Apoe^-/-^ mice (21). To the best of our knowledge, this is the first study to demonstrate this phenomenon in humans.

Through statistical analysis, we founded that patients with enlarged LNs had lower BMI and lower body weight. Some scholars have conducted a retrospective study on female mammography (31) and founded that the longitudinal and transverse diameters of axillary LN increased with increasing BMI. The author attributed the increase in LN size to expansion of the LN hilum caused by fat infiltration. This adds to the evidence that enlarged pericarotid LNs are associated with plaque formation rather than obesity. Meanwhile, lower systolic blood pressure in patients without enlarged LNs may partly explain the stiffer aorta in these patients (32); however, the differences in systolic blood pressure and pulse pressure were not statistically significant. Calcium channel blockers (CCB) are commonly used in patients with enlarged LNs. However, a literature review found no evidence of a relationship between CCB and enlarged LNs. Therefore, our result may not be of practical significance.

Macroscopic examination revealed that the non-enlarged LN was round or oval in shape, but the enlarged LN remained oval in shape, and the hilum was not obvious. Microscopic observation revealed that LN enlargement mainly result from an increase in the number of cells. First, an increase in the number of cells in the LNs may result from an increase in the influx of cells into the LNs. Lymphatic vessels carrying various types of immune cells have been found in the adventitia of atherosclerotic arteries (33). The establishment and development of the lymphatic system can reduce the deposition of lipids and immune cells in arteries (34). These immune cells travel along the lymphatic vessels into the draining LNs, resulting in increased cell numbers in the LNs. Second, this may result from increased cell proliferation in LNs. We observed an increased proportion of Ki67-positive cells in the LN cortex. Although there was no difference in the proportion of Ki67-positive cells in the LN GCs, the number and volume of GCs increased. Finally, impairment of lymphocyte export also contributed to increased cell numbers in the LNs. Tay et al. suggested that disruption of lymphocyte output and interception signals in Apoe^-/-^ mouse LNs leads to the deposition of lymphocytes, resulting in LNs enlargement (35). In our study, CD20^+^ B cells and CD3^+^ T cells were found to accumulate in the cortex and subcapsular sinuses of the LNs. In summary, increased cellular input, decreased output, and enhanced cellular proliferation contribute to the appearance of enlarged LNs.

GCs are transient histological microstructures formed within the follicles of secondary lymphoid tissues in response to foreign pathogens (36). GCs are the sites of B cell selection and maturation and play a vital role in the activation and development of immune system function (37). In our study, we found that the number and volume of GCs in enlarged LNs were higher than those in non-enlarged LNs. At the same time, a large number of proliferating B cells in the GC also suggest that a strong immune response was occurring around the carotid artery in patients with enlarged LNs. B cells are found in the adventitia of atherosclerotic arteries (38, 39), and these cells even form small lymphoid follicles (40, 41). The transfer of spleen B cells into splenectomized Apoe^-/-^ mice alleviates the development of atherosclerosis (42), suggesting that B cells, and consequently humoral immunity, have a protective effect against atherosclerosis. In summary, we think that enlarged pericarotid LNs indicate robust antiatherogenic response.

T cells, another important immune cell in adaptive immunity, are key modulators of atherosclerosis. T cell subpopulations differ at different stages of atherosclerosis, in both plaque and circulation (43). Furthermore, different subpopulations of T cells have different effects on atherosclerosis progression through immune activation, immune suppression, or by helping B cells produce antibodies (44). T cells in pericarotid LNs may play a supporting role, but the specific role still needs to be further explored.

Macrophages play a key role in atherosclerosis progression. Monocyte-derived macrophages phagocytize lipoproteins and develop into lipid-rich foam cells, resulting in the formation of a necrotic core (45, 46). Consistent with previous reports (47), we also found macrophage infiltration in the shoulder region of atherosclerotic plaque. But there is no significant difference between the two (**Figures 2I–L**). Ox-LDL deposited in the intima of artery initiates immunity by interacting with macrophages to remove local cell debris, produce cytokines, and promote local inflammatory responses (48). However, lymphatic reverse cholesterol transport (RCT), which is carried by macrophages, reduces the accumulation of cholesterol in arteries (5). Macrophages play a bidirectional regulatory role in atherosclerosis. In our study, most macrophages are found in the lymphatic sinuses, including subcapsular sinus, cortical sinus, and medullary sinus. These macrophages may come from the draining lymphatic vessels, or they may settle here themselves (9). Recent single-cell sequencing revealed an unanticipated function of LN endothelial cells in scavenging ox-LDL (14). Phagocytosis may occur in pericarotid LNs, in which lymphatic endothelial cells remove modified LDLs unloaded by the macrophages.

This study has several limitations. First, it was not a prospective study. Thus, the chronological relationship between the enlarged LNs and ischemic events could not be determined. Second, the mechanistic pathways of the association between enlarged LNs and plaque development were not assessed. Therefore, no causal relationships were observed. Third, the present analysis only looked at the correlation between enlarged LNs and ischemic symptoms, but did not mention complication and follow-up. Fourth, the criteria for enlarged LNs are mainly applicable to tumors. More precise and specialized criteria are required for carotid atherosclerotic diseases. Finally, the sample size may limit the generalizability of the results.

In conclusion, the current study demonstrated that enlarged pericarotid LNs suggest recent ischemic symptoms and may be a sign of plaque destabilization in patients with carotid atherosclerosis. Adaptive immune responses are activated and reinforced in enlarged pericarotid LNs that drain carotid plaque. Further studies are necessary to explore the potential mechanism between pericarotid LNs and plaque stability and to understand the lymphatic system as a potential therapeutic target in patients with carotid atherosclerosis.

## Data Availability Statement

The original contributions presented in the study are included in the article/supplementary material. Further inquiries can be directed to the corresponding authors.

## Ethics Statement

The studies involving human participants were reviewed and approved by the Ethics Committee of the Qilu Hospital, Shandong University. The patients/participants provided their written informed consent to participate in this study.

## Author Contributions

TS, XL and DW conceived and designed the study. TS, PZ, FW, YH, MH, HL and DW were responsible for patient care and treatment, clinical oversight, and clinical data collection. TS, HL and BM collected and characterized samples. TS and FW conducted data analysis. TS and DW wrote the manuscript. XL and DW modified and revised the manuscript. PZ, XL, and DW supervised the study. All authors contributed to the article and approved the submitted version.

## Funding

This work was supported by the Clinical Practical New Technology Development Foundation of Qilu Hospital (grant 2019-7), and the crosswise tasks (contract number: 11691806 and 6010120062).

## Conflict of Interest

The authors declare that the research was conducted in the absence of any commercial or financial relationships that could be construed as a potential conflict of interest.

## Publisher’s Note

All claims expressed in this article are solely those of the authors and do not necessarily represent those of their affiliated organizations, or those of the publisher, the editors and the reviewers. Any product that may be evaluated in this article, or claim that may be made by its manufacturer, is not guaranteed or endorsed by the publisher.
